# Genotoxic and Anti-Genotoxic Assessments of Fermented *Houttuynia cordata* Thunb. Leaf Ethanolic Extract and Its Anti-Cancer Effect in a Dual-Organ Carcinogenesis Model of Colon and Liver in Rats

**DOI:** 10.3390/foods13223645

**Published:** 2024-11-15

**Authors:** Chonikarn Singai, Pornsiri Pitchakarn, Sirinya Taya, Rawiwan Wongpoomchai, Ariyaphong Wongnoppavich

**Affiliations:** 1 Department of Biochemistry, Faculty of Medicine, Chiang Mai University, Chiang Mai 50200, Thailand; chonikarn.s@gmail.com (C.S.); pornsiri.p@cmu.ac.th (P.P.); rawiwan.wong@cmu.ac.th (R.W.); 2 Functional Food Research Unit, Multidisciplinary Research Institute, Chiang Mai University, Chiang Mai 50200, Thailand; sirinya.t@cmu.ac.th

**Keywords:** diethylnitrosamine, 1,2-dimethylhydrazine, cancer prevention, *Salmonella* mutation assay, micronucleus test

## Abstract

The incidence of multiple-organ cancers has recently increased due to simultaneous exposure to various environmental carcinogens. *Houttuynia cordata* Thunb. (*H. cordata*) is recognized for its many health benefits, including its anti-cancer properties. The fermentation of its leaves has been shown to significantly enhance the bioflavonoid content and its bioactivities. This study aimed to evaluate the effectiveness of fermented *H.cordata* leaf (FHCL) extracts against combined carcinogens and investigate the underlying mechanisms. The crude ethanolic extract of FHCL was partitioned to obtain hexane- (HEX), dichloromethane- (DCM), ethyl acetate- (ETAC), butanol- (nBA), and residue fractions. The crude ethanolic extract (200–250 μg/mL) and the DCM fraction (50 μg/mL) significantly reduced NO production in RAW264.7 macrophages. In addition, the crude extract and the DCM and ETAC fractions showed anti-genotoxicity against aflatoxin B_1_ and 2-amino-3,4-dimethylimidazo [4,5-f]quinoline (MeIQ) in *Salmonella typhimurium* assays (S9+). Despite demonstrating genotoxicity in the *Salmonella* mutation assay (with and without S9 activation), oral administration of the crude extract at 500 mg/kg of body weight (bw) for 40 days in rats did not induce micronucleated hepatocytes, indicating that the extract is non-genotoxic in vivo. Moreover, the crude extract significantly decreased Phase I but increased Phase II xenobiotic-metabolizing enzyme activities in the rats. Next, the anti-cancer effects of FHCL were evaluated in a dual-organ carcinogenesis model of the colon and liver in rats induced by 1,2-dimethylhydrazine (DMH) and diethylnitrosamine (DEN), respectively. The crude extract significantly reduced not only the number and size of glutathione *S*-transferase placental form positive foci in the liver (at doses of 100 and 500 mg/kg bw) but also the number of aberrant crypt foci in rat colons (at 500 mg/kg bw). Furthermore, FHCL significantly reduced the expression of proliferating cell nuclear antigen (PCNA) in the colon (at 100 and 500 mg/kg bw) and liver (at 500 mg/kg bw) of the treated rats. In conclusion, FHCL exhibits significant preventive properties against colon and liver cancers in this dual-organ carcinogenesis model. Its mechanisms of action may involve anti-inflammatory effects, the prevention of genotoxicity, the modulation of xenobiotic-metabolizing enzymes, and the inhibition of cancer cell proliferation. These findings support the use of FHCL as a natural supplement for preventing cancer.

## 1. Introduction

Multiple primary cancers are defined as the synchronous or metachronous occurrence of two or more primary malignant tumors in the same individual, with an increasing incidence reaching up to 17%, making it a significant concern in oncology [1,2]. The colon and liver are major sites for multiple primary cancers of the digestive system, with incidences of 48.9% and 15.2%, respectively [3]. The occurrence of a first primary colorectal cancer is frequently associated with the development of a second primary cancer, often in the colon and liver. Similarly, primary liver cancer usually leads to secondary primary cancers in organs such as the lung, head and neck, and colorectal regions [4,5]. Environmental and lifestyle factors contribute to more than 90% of cancer incidences [1,6,7], with exposure to carcinogens being a significant risk for the development of multiple cancers [8,9]. Carcinogens like heterocyclic amines, dimethylhydrazine, nitrosamines, and aflatoxin B_1_ (AFB_1_) are known to promote colorectal and liver cancers [10,11,12,13]. These carcinogens are metabolized by Phase I xenobiotic enzymes into reactive metabolites that likely cause mutations and initiate oxidative and inflammatory responses, promoting tumor development by enhancing genotoxicity and cell proliferation, and inhibiting apoptosis [14,15,16,17]. Modulating these metabolic enzymes and cellular processes is a promising strategy for cancer prevention, especially for primary cancers induced by environmental carcinogens. However, applying these strategies to prevent multiple cancers is challenging. Natural products often show diminished efficacy in multi-organ cancer models compared to single-carcinogen treatments [18,19,20,21,22]. This highlights the complexity of understanding their full effects in more realistic models of cancer prevention. 

*Houttuynia cordata* Thunb. (*H. cordata*), a medicinal plant widely used in Asia and Southeast Asia [23], emerges as a promising candidate for cancer prevention. Traditionally, it has been used to treat respiratory, digestive, and skin conditions. Previous studies have demonstrated that the extracts of *H. cordata* can suppress Phase I enzyme expression in high-fat diet rats, prevent genetic mutations induced by mutagens in *Salmonella* bacteria, and reduce inflammatory cytokine in lipopolysaccharide (LPS)-induced RAW 264.7 cells [24,25,26]. Moreover, extracts of *H. cordata* have shown anti-cancer activity against colon (HT29) and liver (HepG2) cancer cell lines and have been found to suppress human hepatocellular carcinoma growth in nude mice models [27,28,29]. It is noteworthy that this plant contains bioactive flavonoids, including rutin, hyperoside, quercetin, and quercitrin [30,31]. These flavonoids have been reported to exert various biological activities, including antioxidant, anti-inflammatory, and anti-cancer effects [32,33].

Fermentation is a traditional technique used in traditional medicine to enhance herbal efficacy and reduce the adverse effects of herbal medicines [34,35]. Notably, the fermentation of *H. cordata* leaf with *Bacillus* strains has been shown to enhance the content of key bioactive flavonoids, including rutin, quercetin, and quercitrin, which increase its anti-cancer properties [36]. The bacterial fermented *H. cordata* leaf has shown significant anti-cancer effects by inducing apoptosis and inhibiting cell proliferation in colon and liver cancer cell lines, as well as by reducing inflammation [37,38,39]. Overall, fermented *H. cordata* leaf (FHCL) shows promising potential as effective cancer chemopreventive agents.

Despite these favorable findings, there is still a significant gap in understanding the chemopreventive effects of FHCL in animal models, particularly in models of multiple primary cancers induced by environmental carcinogens. Therefore, this study aims to investigate the effects of the ethanolic extract of FHCL in a dual-carcinogenesis model involving 1,2-dimethylhydrazine (DMH)-induced colon and diethylnitrosamine (DEN)-induced liver carcinogenesis. Moreover, this study explores the possible mechanisms of cancer preventive effects, including the modulation of xenobiotic enzymes, anti-inflammatory activity, and anti-genotoxic properties.

## 2. Materials and Methods

### 2.1. Chemicals and Reagents

Lipopolysaccharides (LPS) from *Escherichia coli O111*, Griess reagent, and 3-(4,5-Dimethylthiazol-2-yl)-2,5-Diphenyltetrazolium Bromide (MTT) reagent were obtained from Sigma-Aldrich (St. Louis, MO, USA). Resorufin, ethoxyresorufin, uridine 5′- diphosphate -glucuronic acid (UDP-GA), para-nitrophenol (*p*-NP), and 3,3′-diaminobenzidine (DAB) were also sourced from Sigma-Aldrich. Standard mutagens, including aflatoxin B1 (AFB_1_), sodium azide (NaN_3_), and diethylnitrosamine (DEN), were purchased from the same supplier. Additional mutagens, such as 2-aminoanthracene (2-AA), 2-(2-furyl)-3-(5-nitro-2-furyl)-acrylamide (AF-2), and 2-amino-3,4-dimethylimidazo[4,5-f]quinoline (MeIQ), along with the substrate methoxyresorufin, were obtained from Wako Pure Chemicals (Osaka, Japan). Dimethylhydrazine (DMH) was sourced from the Tokyo Chemical Industry (Tokyo, Japan). Collagenase type IV and 4′,6-diamidino-2-phenylindole (DAPI) were acquired from Invitrogen Corp. (Carlsbad, CA, USA). Additionally, 1-chloro-2,4-dinitrobenzene (CDNB) and bovine serum albumin (BSA) were purchased from Thermo Fisher Scientific Inc. (Waltham, MA, USA). Dulbecco’s Modified Eagle Medium (DMEM), penicillin-streptomycin, and fetal bovine serum (FBS) were obtained from Gibco/Invitrogen Crop. (Grand Island, NY, USA). The avidin–biotin–horseradish peroxidase complex (ABC) kit and Vectastain Elite ABC kit (Universal) were obtained from Vector Laboratories Inc. (Burlingame, CA, USA), while the Apoptag detection kit was purchased from Merck Millipore (Darmstadt, Germany). All other chemicals used in this study were of analytical or HPLC grade.

### 2.2. Preparation of Fermented Houttuynia cordata Leaf (FHCL) Extract and Its Fractions

*Houttuynia cordata* Thunb. (Queen Sirikit Botanic Garden Herbarium: QBG. No. 131046) was cultivated and harvested in Chai Badan District, Lopburi, Thailand. The harvested plants underwent fermentation and quality control procedures according to the guidelines and regulations managed by Prolac (Thailand) Co., Ltd. (Lamphun, Thailand). In brief, approximately 400 kg of *H. cordata* leaf was harvested and fermented with 70 kg of sugarcane powder, 150 L of in-house bacteria culture, and 800 L of water [40]. Prolac Co., Ltd. generously provided the fermented *Houttuynia cordata* leaf (FHCL) powder, which was extracted twice with 70% ethanol over two days and then freeze-dried to obtain the crude extract. This extract was dissolved in deionized (DI) water for in vivo studies.

The crude extract was then partitioned sequentially using hexane, dichloromethane, ethyl acetate, and butanol in a 1:1 ratio, yielding hexane (HEX), dichloromethane (DCM), ethyl acetate (ETAC), butanol (nBA), and residue fractions. All fractions were evaporated, freeze-dried, and dissolved in dimethyl sulfoxide (DMSO) for in vitro studies.

### 2.3. The Determination of Phytochemicals in the Crude Extract and Its Fractions

The contents of total phenolics, total flavonoids, and total hydrolysable tannins of crude extract and its fractions were analyzed spectrophotometrically using the Folin–Ciocalteu colorimetric method, aluminum chloride colorimetric method, and potassium iodate method, respectively [41,42]. The HPLC technique was employed to analyze specific phenolics and flavonoids in all fractions using reverse-phase HPLC with an Agilent ZORBAX Eclipse Plus C18 column (4.6 × 250 mm, 5 μM) (Agilent Technologies, Santa Clara, CA, USA) as described in a previous study [43]. The concentration of each component in the sample was quantified using a standard curve for each standard compound, including gallic acid, protocatechuic acid, 4-hydroxybenzoic acid, chlorogenic acid, vanillic acid, syringic acid, p-coumaric acid, ferulic acid, ellagic acid, *trans*-cinnamic acid, catechin, epicatechin, rutin, quercetin, luteolin, apigenin, quercitrin, myricetin, naringenin, kaempferol, and isorhamnetin.

### 2.4. Cell Culture and Culture Condition

RAW264.7 macrophage cells (ATCC TIB-71, Manassas, VA, USA) were cultured in DMEM supplemented with 10% fetal bovine serum (FBS) and 1% penicillin-streptomycin. The cells were maintained at 37 °C in a humidified atmosphere with 5% CO_2_.

### 2.5. A Cytotoxicity Assessment of the Crude Extract and Its Fractions on RAW 264.7 Macrophages

The cytotoxicity of the crude extract and its fractions on RAW 264.7 macrophages was assessed using an MTT assay [44]. RAW 264.7 cells were seeded at a density of 2 × 10^5^ cells/mL and treated with test samples at concentrations of up to 500 μg/mL for 24 h. Following the treatment, the cells were incubated with the MTT reagent at a final concentration of 0.5 mg/mL for two hours to assess cell viability. The formazan crystal was then dissolved with dimethyl sulfoxide (DMSO). The absorbance of the solubilized formazan was then measured at 540 nm and 630 nm. The results were reported as IC_20_, and a concentration below IC_20_ was considered non-cytotoxic. Concentrations of the fractions that did not exceed their IC_20_ values were used for further investigation of their potential effects on LPS-induced NO production in RAW 264.7 macrophages.

### 2.6. The Determination of the Anti-Inflammatory Activity of the Crude Extract and Its Fractions

The anti-inflammatory activity of the crude extract and its fractions was determined using a nitric oxide (NO) scavenging assay [44]. RAW 264.7 cells were seeded at a density of 2 × 10^5^ cells/mL and treated with various concentrations of the fractions. After two hours of treatment, the cells were exposed to 1 μg/mL of LPS for 24 h, and then the supernatant was collected to evaluate the NO concentration using Griess reagent. The NO concentration was measured at 550 nm and quantified using a sodium nitrite standard curve. The results were expressed as the relative NO production to the LPS-induced NO alone.

### 2.7. An Evaluation of the In Vitro Genotoxicity and Anti-Genotoxicity of the Crude Extract and Its Fractions Using an Ames Test

The genotoxicity and anti-genotoxicity tests of the crude extract and its fractions were performed using *Salmonella typhimurium* strains TA 98 and TA100 in both the presence (+S9) and absence (−S9) of metabolic activation as described previously [45]. The bacterial tester strains were kindly provided by Dr. Kei-Ichi Sugiyama, National Institute of Health, Tokyo, Japan.

For the genotoxicity assay, various concentrations of each fraction were mixed with the bacteria in the presence (+S9) or absence (−S9) of the S9 mixture. This mixture was incubated at 37 °C for 20 min in a shaking water bath. Subsequently, top agar containing L-histidine and D-biotin was added and poured onto minimal agar plates, which were then incubated at 37 °C for 48 h. The mutagens 2AA and AF-2 were used as positive controls in the presence and absence of metabolic activation, respectively. The number of revertant colonies was counted and expressed as a mutagenic index (MI), which is the ratio of the number of revertant colonies to the control. A sample was identified as mutagenic if the MI exceeded two.

For the anti-genotoxicity test, non-cytotoxic doses of each fraction were mixed with the bacteria, mutagen, and either the +S9 or −S9 buffer. The mixture was incubated at 37 °C for 20 min and then poured onto minimal agar plates, followed by further incubation at 37 °C for 48 h. AFB_1_ and MeIQ were used as indirect mutagens for TA98 and TA100, respectively, under metabolic activation conditions. AF-2 and NaN_3_ were used as direct mutagens for TA98 and TA100, respectively, under conditions without metabolic activation. Anti-genotoxic activity was expressed as the average number of His^+^ revertant colonies per plate and as a percentage of inhibition.

### 2.8. Animals

All Wistar rats used in this study were obtained from Nomura Siam International Co., Ltd. in Bangkok, Thailand. They were housed in the Animal House at the Faculty of Medicine, Chiang Mai University, under standard environmental conditions throughout the experiments. The oral dosage was determined from the results of an acute oral toxicity test, which was conducted following OECD guideline No. 425 [46]. The experimental protocols for rats were approved by the Animal Ethics Committee of the Faculty of Medicine, Chiang Mai University (Protocol No. 07/2563, approved on 17 April 2020, and Protocol No. 38/2563, approved on 26 July 2020). All procedures were performed in compliance with relevant guidelines and regulations, adhering to the recommendations outlined in the ARRIVE guidelines.

### 2.9. In Vivo Genotoxicity Assessment Using Micronucleus Test

The genotoxicity of crude FHCL was assessed in vivo by the micronucleus test, following a modified method previously described in [47]. Four-week-old male Wistar rats were randomly divided into two groups, with five rats per group. Group 1 received deionized water and served as the negative control, while Group 2 was fed crude extract by oral gavage at a concentration of 500 mg/kg of body weight (bw) for 40 days. On day 41, all rats underwent partial hepatectomy to accelerate the proliferation of mutated cells. On day 43, the rats were anesthetized, and hepatocytes were isolated using a two-step collagenase perfusion method as described previously [48]. The isolated hepatocytes were stained with DAPI and counted under a fluorescent microscope (Olympus AX70, Tokyo, Japan). Micronucleated cells were scored according to the criteria described by Cliet et al. [49].

### 2.10. Measurement of Xenobiotic-Metabolizing Enzyme Activities in the Livers of Rats Receiving Crude FHCL

The remaining rat liver samples from Section 2.9 were homogenized to prepare cytosolic and microsomal lysates by following a previously described method [50]. The protein concentrations of these lysates were measured using the Lowry method in the comparison of a standard curve of bovine serum albumin [51].

The activities of cytochrome P450 (CYP), 1A1, and 1A2 were assessed using ethoxyresorufin-O-deethylation (EROD) and methoxyresorufin-O-demethylation (MROD) assays, respectively. Each substrate was mixed with isolated liver microsomes, NADPH, and magnesium chloride. The production of resorufin, the end-product of both assays, was measured by Agilent BioTek Synergy H4 Hybrid Microplate Reader (Winooski, VT, USA) at excitation and emission wavelengths of 520 nm and 590 nm, respectively. The enzyme activities were calculated from the linear equation of a resorufin standard curve and expressed as units per mg of protein [52].

The activity of CYP2E1 was measured by quantifying *p*-nitrocatechol formation from *p*-nitrophenol (*p*-NP). The liver microsomes were pre-incubated with *p*-NP, NADPH, magnesium chloride, D-glucose-6 phosphate, and glucose-6 phosphate dehydrogenase. The reaction was then stopped with 10% TCA and centrifuged at 10,000 g for 5 min. The supernatant was neutralized with NaOH, and the end-product, 4-nitrocatechol, was monitored by Agilent BioTek Synergy H4 Hybrid Microplate Reader (Winooski, VT, USA) at 535 nm. The CYP2E1 activity in each sample was calculated from the linear equation generated by a 4-nitrocatechol standard curve and expressed as units per mg of protein [50].

The activity of CYP3A2 was measured using the erythromycin N-demethylation (ENDM) assay. CYP3A2 activity was determined using an Agilent BioTek Synergy H4 Hybrid Microplate Reader (Winooski, VT, USA), based on a formaldehyde standard curve at a wavelength of 405 nm, and expressed as units per mg of protein [52].

Glutathione S-transferase (GST) activity was assessed using 1-chloro-2,4-dinitrobenzene (CDNB) as a substrate. Liver cytosol was added to a reaction mixture containing phosphate buffer and glutathione. After 90 s, the absorbance of the CDNB-GSH conjugate was measured by Agilent BioTek Synergy H4 Hybrid Microplate Reader (Winooski, VT, USA) at 340 nm. The activity was calculated using a molar extinction coefficient of 9.6 M^−^^1^ cm^−^^1^ and expressed as units per mg of protein [52].

UDP-glucuronosyl transfersase (UGT) activity was analyzed by measuring the conjugation of *p*-NP. Liver microsomes were pre-incubated with a reaction mixture consisting of Tris, magnesium chloride, *p*-NP, and UDP-glucuronic acid (UDP-GA). The reaction was stopped with 10% TCA, followed by centrifugation at 10,000 rpm for 5 min. The supernatant was mixed with NaOH, and the conjugated *p*-NP level was quantified by Agilent BioTek Synergy H4 Hybrid Microplate Reader (Winooski, VT, USA) at 405 nm and calculated using a molar extinction coefficient of 9.6 M^−^^1^ cm^−^^1^ and expressed as units per mg of protein [52].

### 2.11. Anti-Carcinogenicity Assessment of Crude FHCL Using a Dual-Organ Model in Rats

The anticarcinogenic effects of crude FHCL ethanolic extract were examined using a dual-organ carcinogenicity model following a methodology described previously [18,53]. Four-week-old male rats were randomly assigned to six groups, each comprising eight rats. To induce colorectal carcinogenesis, Groups 4 to 6 were injected subcutaneously (s.c.) with 40 mg/kg bw of DMH on days 7 and 14. These groups also intraperitoneally (i.p.) received 100 mg/kg bw of DEN on days 7, 11, and 18 to induce hepatocarcinogenesis. Conversely, Groups 1 to 3 were administered injections of 0.9% normal saline (NSS) either subcutaneously or intraperitoneally instead of DMH and DEN, respectively.

For the treatment, Groups 1 and 4, serving as vehicle controls, were administered deionized water via gavage five days a week. Groups 2 and 5 received 100 mg/kg bw of the crude extract via gavage five days a week, whereas Groups 3 and 6 received 500 mg/kg bw of the crude extract via gavage. At the end of the ten-week study, the rats were humanely euthanized. Blood samples were collected for serum alanine aminotransferase (ALT) level measurement, which was conducted by the Small Animal Hospital at Chiang Mai University, Thailand. Additionally, liver and colon tissues were collected to evaluate preneoplastic lesions and to investigate the expression of cell proliferation- and death-associated markers.

### 2.12. Determination of Preneoplastic Lesions in Liver and Colon Tissues

Hepatic preneoplastic lesions were identified by detecting glutathione *S*-transferase placental form (GST-P)-positive foci using an immunohistochemical staining technique [54]. Initially, liver sections were deparaffinized and immersed in a solution containing 3% hydrogen peroxide (H_2_O_2_) and skimmed milk to block endogenous peroxidase activity and reduce non-specific binding, respectively. Following this, the slides were incubated with a rabbit polyclonal anti-rat GST-P antibody (MBL, Nagoya, Japan). An avidin–biotin complex (ABC) kit and the substrate diaminobenzidine (DAB) were then used to develop brown coloration, followed by counterstaining with hematoxylin. The numbers and sizes of GST-P-positive foci were quantified using the Leica Application Suite (LAS) Interactive Measurement program (Leica Microsystems, Wetzlar, Germany).

Colonic preneoplastic lesions were identified by staining aberrant crypt foci (ACF) with methylene blue [54]. Formalin-fixed colons were immersed in a 2% methylene blue solution for staining. The total number of aberrant crypts per rat and the number of aberrant crypts per focus were then evaluated using a light microscope (Nikon Corporation, Tokyo, Japan).

### 2.13. Determination of Cell Proliferation Marker Using Immunohistochemical Staining

Immunohistochemical staining for proliferating cell nuclear antigen (PCNA), a cell proliferation marker, was conducted following a previously described method [18]. Liver and colon sections were first deparaffinized and then treated with 10 mM citrate buffer (pH 6.0) using autoclaving for antigen retrieval. The sections were then incubated with monoclonal mouse anti-rat PCNA antibodies (BioLegend, San Diego, CA, USA), followed by treatment with biotinylated secondary antibodies. An Elite avidin–biotin complex kit was used in combination with DAB for visualization, with hematoxylin serving as a counterstain. The quantification of PCNA-positive cells was performed using a light microscope (Leica Microsystems, Wetzlar, Germany).

### 2.14. Determination of Apoptotic Cells Using Terminal Deoxynucleotidyltransferase (TdT)–dUTP Nick End Labeling (TUNEL) Assay

Apoptotic cells in the liver and colon were identified using an ApopTag Peroxidase in situ kit as previously described by Thumvijit et al., 2014 [55]. The procedures were carried out according to the kit instructions, with color development achieved through the application of DAB. The number of apoptotic cells was then counted using a light microscope (Leica Microsystems, Wetzlar, Germany).

### 2.15. Statistics Analysis

Data are presented as means ± standard deviation (SD). Statistical comparisons between groups in the micronucleus test were performed using the independent Student’s *t*-test. For other analyses, a one-way analysis of variance (ANOVA) was conducted, followed by a Bonferroni post hoc analysis using GraphPad Prism 9.0 software (GraphPad Software, Boston, MA, USA). Differences were considered significant when *p* ≤ 0.05. The significance level for each result is indicated in the corresponding graphs and tables.

## 3. Results

### 3.1. Phytochemical Components in FHCL and Its Fractions

The ethanolic extract of FHCL was sequentially partitioned, yielding hexane (HEX), dichloromethane (DCM), ethyl acetate (ETAC), butanol (nBA), and residue fractions, with the percentage yields detailed in Table 1. The residue had the highest yield, followed by nBA, HEX, ETAC, and DCM, respectively. A spectrophotometric analysis showed that the crude FHCL extract was rich in total phenolics, flavonoids, and hydrolysable tannins, with 21.09, 16.58, and 4.05 mg/g of dried powder, respectively. Among the fractions, ETAC had the highest phenolic (25.33 mg/g of crude) and flavonoid (23.25 mg/g of crude) contents, while both nBA (11.43 mg/g of crude) and ETAC (11.33 mg/g of crude) had the highest levels of total hydrolysable tannins. These results suggest that flavonoids were the predominant polyphenols in the crude extract and its fractions. An HPLC analysis further revealed that the crude extract contained flavonoids such as rutin (0.644 mg/g of dried powder), quercetin (0.088 mg/g of dried powder), and quercitrin (0.050 mg/g of dried powder), with quercitrin being the most abundant. In contrast, phenolics including protocatechuic acid, chlorogenic acid, vanillic acid, and p-coumaric acid were present in lower amounts and were below detectable levels using HPLC. Among the fractions, protocatechuic acid (1.741 mg/g of crude), chlorogenic acid (2.897 mg/g of crude), and rutin (4.934 mg/g of crude) were noted for their high concentrations in the ETAC fraction. Yet, the DCM fraction was rich in vanillic acid (0.048 mg/g of crude), p-coumaric acid (0.508 mg/g of crude), and quercetin (2.153 mg/g of crude) (Table 1).

### 3.2. Anti-Inflammatory Activities of FHCL Extract and Its Fractions on Lipopolysaccharide (LPS)-Stimulated RAW 264.7 Macrophage Cell Lines

**Figure 1:**
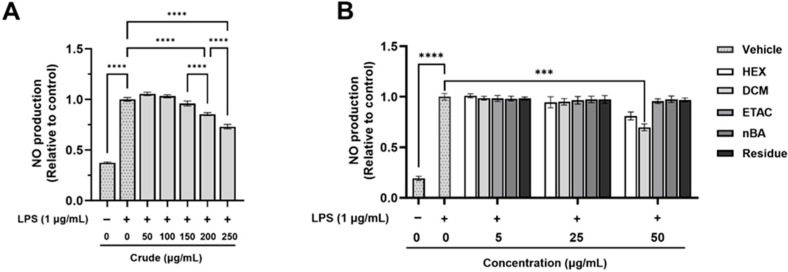
The effect of FHCL crude ethanolic extract (**A**) and its fractions (**B**) on nitric oxide (NO) production in lipopolysaccharide (LPS)-stimulated RAW 264.7 macrophages was assessed using Griess reagents. The results are presented as the mean ± standard deviation from three independent experiments. Significance levels are indicated as *** *p* ≤ 0.001 and **** *p* ≤ 0.0001. HEX: hexane fraction; DCM: dichloromethane fraction; ETAC: ethyl acetate fraction; nBA: butanol fraction.

The anti-inflammatory effects of the crude extract and its fractions were evaluated using non-toxic doses (Table S1) on LPS-induced nitric oxide (NO) production in RAW 264.7 macrophages. The stimulation of RAW 264.7 macrophage cells with LPS led to a significant increase in NO production compared to non-LPS-treated cells (Figure 1). However, pre-treatment with the crude extract at 200 and 250 µg/mL significantly reduced LPS-induced NO production by 14.55% and 26.89%, respectively (Figure 1A). The DCM fraction at a concentration of 50 µg/mL also significantly inhibited LPS-induced NO production by 30.20% compared to cells treated with LPS alone; however, the other fractions exhibited no significant changes (Figure 1B). Interestingly, calculations based on the % yield of the crude extract indicate that a 250 µg/mL dose of the crude extract contains approximately 10 µg/mL of the DCM fraction, which is below the effective dose for DCM. The results suggest that the crude extract is more effective than the individual fractions, as it can reduce NO production at much lower concentrations. These findings highlight the strong efficacy of the crude FHCL extract in preventing inflammation by significantly lowering NO production from inflammatory cells. Additionally, both the crude extract and the DCM fraction contain bioactive compounds that contribute to their anti-inflammatory effects.

### 3.3. In Vitro Genotoxic Properties of FHCL Crude Extract and Its Fractions

The genotoxic properties of the crude FHCL extract and its fractions were evaluated using the Ames test (Salmonella mutation assay) in bacteria strains TA98 and TA100 with and without metabolic activation (S9 mix). As shown in Table 2, the results reveal that the crude extract caused a two-fold increase in the number of revertant colonies (MI ≥ 2) at concentrations of 1 and 5 µg/plate in both −S9 and +S9 conditions for TA98, indicating that genotoxicity occurs independently of metabolic activation. In TA100, genotoxicity (MI ≥ 2) was observed only at 5 µg/plate of the crude extract under the +S9 condition, while no genotoxicity (MI < 2) was detected under the −S9 condition. This suggests that genotoxic effects are more pronounced in TA98, particularly under the +S9 condition. Interestingly, the DCM and ETAC fractions exhibited MI ≥ 2 at the lowest concentration (0.2 µg/plate) in TA98 under both − and +S9 conditions, with increasing doses resulting in higher MI values. In TA100 under the +S9 condition, ETAC also exhibited MI ≥ 2 at 0.2 µg/plate, whereas DCM displayed this effect starting at 1 µg/plate. However, the genotoxic effect diminished at lower concentrations (0.2 µg/plate for DCM, and 0.2 and 0.1 µg/plate for ETAC) in TA100 under the −S9 condition. Additionally, the HEX and nBA fractions demonstrated MI ≥ 2 only at the highest concentration (5 µg/plate) in TA98. In contrast, the residue fraction showed no genotoxic properties in TA98 or TA100 under both − and +S9 conditions. These findings suggest that all fractions, except the residue, can cause genotoxicity in Salmonella bacteria regardless of the requirement for metabolic activation, with more pronounced effects at higher doses. Next, these genotoxic effects were further validated by performing subsequent animal experiments.

### 3.4. Anti-Genotoxicity Properties of FHCL Crude Extract and Its Fraction in Salmonella typhimurium

The anti-genotoxic properties of the FHCL crude extract and its fractions were evaluated using *Salmonella typhimurium* against standard mutagens (Table 3). In the presence of metabolic activation (+S9), significant reductions in revertant colonies of both TA98 and TA100 exposed to aflatoxin B_1_ (AFB_1_) and 2-amino-3,4-dimethylimidazo[4,5-f]quinoline (MeIQ) were observed in the groups treated with the crude extract and many fractions, including HEX, DCM, and ETAC, at concentrations ≥ 0.2 mg/plate, indicating anti-genotoxic effects against indirect mutagens. However, their effectiveness likely declined at higher concentrations. The nBA fraction also showed anti-genotoxicity against AFB_1_ and MeIQ-induced mutagenesis at concentrations ≥ 1 mg/plate, while the residue fraction had no effect. Notably, the DCM fraction exhibited significant reductions in revertant colonies in both strains of bacteria at a lower concentration (0.04 mg/plate) compared to other fractions, demonstrating the highest anti-genotoxic potential among those tested. However, 0.04 mg/plate of the DCM fraction is equivalent to 1 mg/plate of the crude extract based on the % yield calculation. The crude extract at 1 mg/plate achieved 88.1% inhibition, while the DCM fraction at 0.04 mg/plate showed 66.8% inhibition, highlighting the stronger anti-genotoxic effect of the crude extract over its fractions. In contrast, in the absence of metabolic activation (−S9), none of the FHCL fractions demonstrated anti-genotoxic effects against AF-2 and NaN_3_. These findings suggest that the crude and DCM fractions of FHCL contain anti-mutagenic compounds that are capable of blocking gene mutations induced by indirect mutagens. In addition, these inhibitory effects of the extracts may be attributed to their modulation of xenobiotic-metabolizing enzymes.

### 3.5. In Vivo Genotoxicity Assessment of FHCL Crude Extract

**Figure 2:**
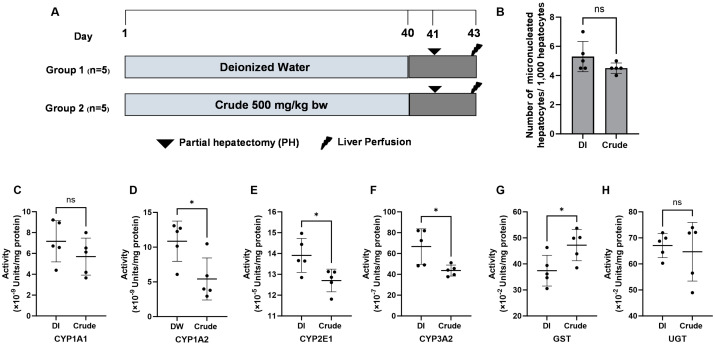
The genotoxicity protocol. (**A**) The effect of crude FHCL ethanolic extract on micronucleated hepatocytes (**B**) and the activities of xenobiotic-metabolizing enzymes were evaluated in rat liver. Deionized (DI) water was used as the negative control. The enzymes studied included Phase I enzymes CYP1A1 (**C**), CYP1A2 (**D**), CYP2E1 (**E**), and CYP3A2 (**F**) and Phase II enzymes glutathione S-transferases (GST) (**G**) and UDP-glucuronosyltransferase (UGT) (**H**). Specific assays were performed for each enzyme. The results are presented as the mean ± standard deviation. Statistical significance was indicated by * (*p* ≤ 0.05) when compared to the vehicle control group. “ns” denotes non-significant differences (*p* > 0.05).

While the FHCL crude extract exhibited the genotoxic potential in bacteria, this study further assessed toxicity using the micronucleus test in an animal model (Figure 2A). The acute toxicity test revealed that the LD_50_ of the crude extract exceeded 5000 mg/kg bw. Based on OECD guideline No. 425, we chose a dose of 500 mg/kg bw (a 10-fold reduced dose) for subsequent experiments. The micronucleus test indicated no significant differences in the number of micronucleated hepatocytes in rats administered 500 mg/kg bw of crude extract compared to the non-treated control groups (Figure 2B), suggesting that the extract is non-genotoxic in vivo.

### 3.6. Effect of FHCL Crude Extract on Xenobiotic-Metabolizing Enzymes Activities in Rat Livers

Xenobiotic-metabolizing enzymes are essential for detoxifying and eliminating harmful foreign compounds, known as xenobiotics; however, some metabolites of these xenobiotics can inadvertently lead to genotoxicity. Next, we investigated whether the FHCL crude extract alters the activities of Phase I and Phase II xenobiotic-metabolizing enzymes in rat liver. The crude extract at 500 mg/kg bw significantly reduced the activities of Phase I enzymes, including CYP1A2, CYP2E1, and CYP3A2, while decreased activity was also noted for CYP1A1 (Figure 2C–F). In contrast, the crude extract significantly increased the activity of the Phase II enzyme glutathione S-transferases (GST), but not UDP-glucuronosyltransferase (UGT) (Figure 2G,H). The results highlight the potential of the extract to reduce chemical-induced genotoxicity by modulating xenobiotic-metabolizing enzyme activities in the liver.

### 3.7. The Anti-Carcinogenic Potential of FHCL Crude Extract in a Dual-Organ Carcinogenesis Model in Rats

**Figure 3:**
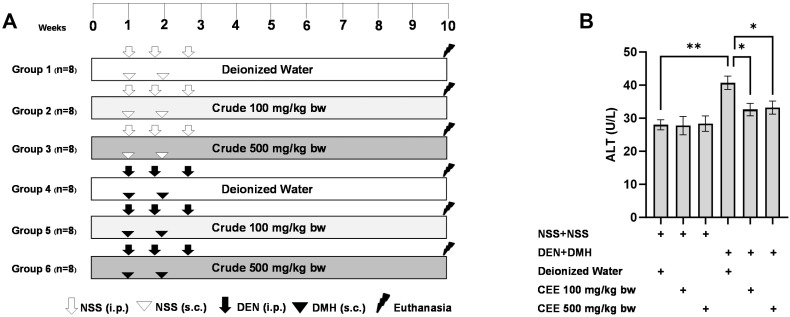
The study protocol for the dual-organ carcinogenesis model, including the timeline for administering carcinogens (DEN and DMH) and crude FHCL extract in rats (**A**). The serum alanine aminotransferase (ALT) levels in rats (**B**). Normal saline (NSS) was used as the control for the carcinogen-treated group, while deionized (DI) water served as the control for the crude extract treatment. The data are presented as the mean ± standard deviation (*n* = 8). Statistical significance is indicated by * (*p* ≤ 0.05) and ** (*p* ≤ 0.01).

**Figure 4:**
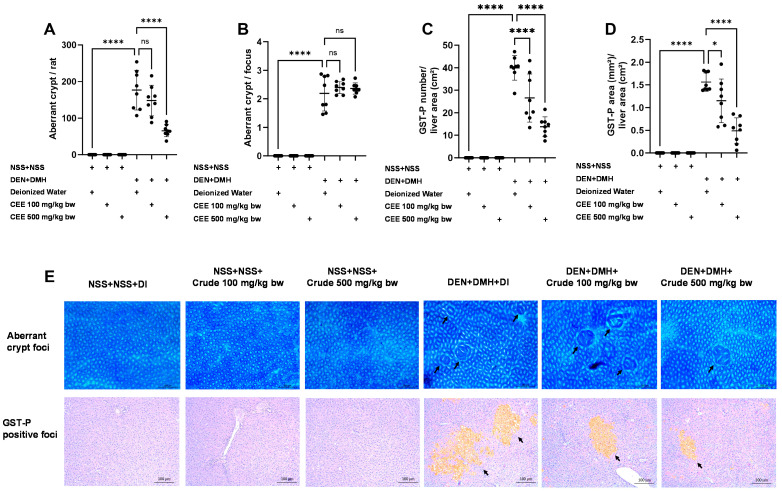
The effects of a ten-week administration period of crude FHCL extract on preneoplastic lesions induced by dual carcinogens in rat colons and livers. The analysis of preneoplastic lesions in the colon includes the number (**A**) and area (**B**) of aberrant crypt foci (ACF), identified with 0.2% methylene blue staining. For the liver, preneoplastic lesions are evaluated by the number (**C**) and size (**D**) of glutathione *S*-transferase placental form (GST-P)-positive foci, detected through immunohistochemistry. The figure shows representative images of preneoplastic lesions (ACF and GST-P-positive foci indicated by arrows) in the colons (40× magnification) and livers (100× magnification) (**E**). The data are presented as the mean ± standard deviation (*n* = 8). Statistical significance is indicated by * (*p* ≤ 0.05) and **** (*p* ≤ 0.0001). “ns” indicates non-significant differences (*p* > 0.05).

Since the crude extract exhibited the most effective anti-carcinogenic effects in vitro, its ability to reduce preneoplastic lesions in rat colons and livers was evaluated using a DMH- and DEN-induced carcinogenesis model (Figure 3A). Exposure to these carcinogens resulted in marked increases in serum alanine aminotransferase (ALT) levels and number of preneoplastic lesions, including aberrant crypt foci (ACF) in the colon and glutathione S-transferase placental form (GST-P) in the liver, compared to the group receiving normal saline (NSS) alone (Figure 3 and Figure 4). Administration of the crude extract at doses of 100 and 500 mg/kg bw in the rats exposed to carcinogens significantly lowered the serum ALT levels compared to the untreated group exposed to the same carcinogens (Figure 3B), indicating a protective effect against carcinogen-induced liver damage. Furthermore, treatment at 500 mg/kg bw significantly reduced the number of colonic aberrant crypts by over 60%, without affecting their size, compared to the control group treated with carcinogens (Figure 4A,B). Interestingly, crude extract at concentrations of 100 and 500 mg/kg bw significantly decreased either the number or the area of GST-P-positive foci by more than half in the liver samples compared to the carcinogen-treated control group (Figure 4C,D). These results suggest that FHCL is effective in controlling the initiation of lesion growth in the colon and liver following carcinogen exposure. However, its effectiveness in preventing the progression of lesions seems to be restricted to the liver during the early stages of carcinogenesis. Importantly, the rats treated with the crude extract at doses of up to 500 mg/kg bw did not cause any spontaneous preneoplastic formation in the colons and livers, indicating that the extract is non-carcinogenic to these organs.

### 3.8. Effect of Crude FHCL Extract on Cell Proliferation and Cell Apoptosis

**Figure 5:**
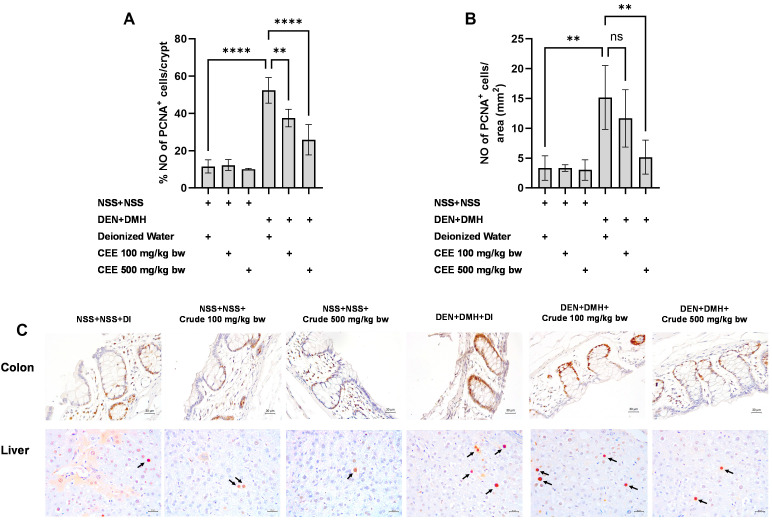
Effect of crude FHCL extract on early-stage carcinogenesis in rats: modulation of cell proliferation. Immunohistochemical staining was used to quantify proliferating cell nuclear antigen (PCNA)-positive cells in colon (**A**) and liver (**B**). Figure displays representative immunohistochemical images of PCNA-positive cells in liver sections (400× magnification) and colon sections (400× magnification) (**C**). In colon, PCNA-positive cells exhibit brownish staining, while PCNA-positive hepatic cells are indicated by arrows. Data are presented as mean ± standard deviation. Statistical significance is denoted by ** (*p* ≤ 0.01) and **** (*p* ≤ 0.0001). “ns” indicates non-significant differences (*p* > 0.05).

**Figure 6:**
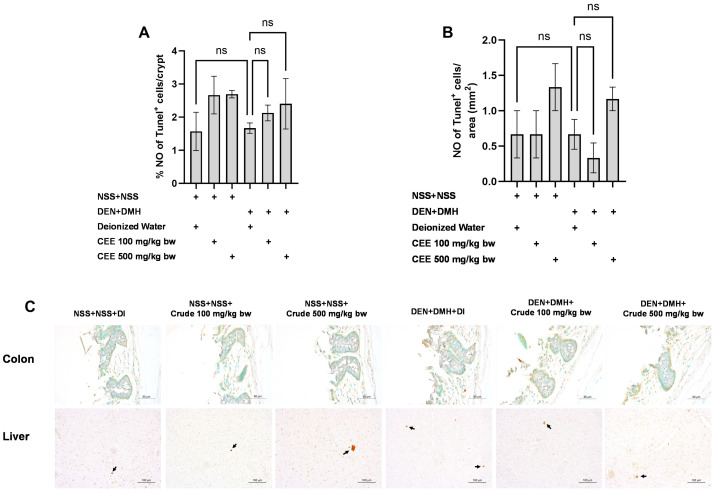
Effect of crude FHCL extract on early-stage carcinogenesis in rats: modulation of cell apoptosis. Immunohistochemical staining was used to quantify TUNEL-positive cells in colon (**A**) and liver (**B**). Figure displays representative immunohistochemical images of TUNEL-positive cells in colon sections (400× magnification) and liver sections (200× magnification) (**C**). TUNEL-positive cells in colon exhibit brownish staining, while TUNEL-positive hepatic cells are indicated by arrows. Data are presented as mean ± standard deviation. “ns” indicates non-significant differences (*p* > 0.05).

We further explored the mechanisms of the extract’s effects on cellular proliferation and apoptosis in the colon and liver. In rats exposed to DMH and DEN, the number of proliferating cell nuclear antigen (PCNA)-positive cells significantly increased by three-fold in both the colon and liver samples compared to the group that received NSS alone, indicating that carcinogen exposure enhances cellular proliferation. The administration of the crude extract at doses of 100 and 500 mg/kg bw significantly reduced the number of PCNA-positive cells in the colons of rats treated with the carcinogens (Figure 5A). In the liver, a marked reduction in PCNA-positive cells was observed when the carcinogen-treated rats received FHCL extract at a dose of 500 mg/kg bw (Figure 5B). However, treatment with the crude extract did not cause significant changes in the number of TUNEL-positive cells, an indicator of apoptosis, in either the colons or livers of rats treated with carcinogens (Figure 6). These findings suggest that FHCL crude extract primarily inhibits the formation and progression of preneoplastic lesions in the early stages of carcinogenesis by affecting cell proliferation rather than apoptosis. Furthermore, treatment with the crude extract alone did not significantly alter cell proliferation or apoptosis in either the colon or liver tissues of normal rats (Figure 5 and Figure 6), indicating that it does not induce spontaneous changes in these two processes.

## 4. Discussion

This research study offers valuable new insights into the potential of fermented *Houttuynia cordata* leaf (FHCL) to prevent multiple primary cancers induced by combined carcinogens in animal models. Using a dual carcinogen-induced rat model, the study shows that crude ethanolic extract markedly reduces preneoplastic lesions in the liver and colon during early carcinogenesis. This effect is mainly due to the modulation of key cellular processes, particularly the inhibition of cell proliferation. Additionally, this study highlights the potential of FHCL in reducing inflammation and inhibiting genotoxic effects by modulating detoxification pathways in Phases I and II.

The genotoxic effects on oncogenes and tumor suppressor genes play a crucial role in the development of carcinogenesis [56]. Flavonoid compounds in FHCL, such as rutin, quercetin, and quercitrin, have been shown to induce genotoxicity in *Salmonella* bacteria models TA98 and TA100 both with and without metabolic activation, with a more pronounced effect being observed in TA98 under +S9 conditions [57,58]. Crude FHCL, as well as its HEX, DCM, ETAC, and nBA fractions, which contain these flavonoids, also exhibited mutagenicity, particularly in TA98 under +S9 conditions. Quercetin and its derivatives interact with DNA through hydrogen bonding and other non-covalent interactions, such as groove binding and intercalation [59,60]. Rutin and quercitrin, due to their glycosylation, show lower genotoxicity compared to quercetin, as their glycosyl groups generate steric hindrance that impedes DNA interaction [61]. These interactions can disrupt the DNA structure, causing replication errors, point mutations, frameshift mutations, or other genetic alterations, especially in rapidly dividing bacterial cells [62,63]. The genotoxic effects were more pronounced in the DCM and ETAC fractions, which have higher concentrations of quercetin, compared to the crude extract. This highlights the advantage of using the crude extract over fractions, as the crude extract may neutralize some of the genotoxic effects of the flavonoids.

Despite these genotoxic effects in bacterial models, the crude FHCL extract and its fractions exhibited significant anti-genotoxic in the same assays. The crude FHCL extract and its fractions, except the residue, could prevent the genotoxic effects of indirect mutagens which induce a frameshift in TA 98 and point mutations in TA 100. This suggests that while these flavonoids may induce genotoxicity, they also have the potential to stabilize DNA and protect it from oxidative damage by reducing DNA accessibility to ROS [64,65,66]. However, the protective effect is concentration-dependent, and higher concentrations of the FHCL and its fractions may reduce their anti-genotoxicity effect, with prolonged exposure potentially causing extensive DNA damage [67]. In contrast to bacterial models, animal studies have shown that quercetin and its derivatives do not exhibit genotoxicity, likely due to their rapid metabolism and lower absorption [68,69]. This highlights the limitations of bacterial models, which lack the complete pharmacokinetic pathways for metabolizing xenobiotic compounds and have less effective DNA repair systems compared to animal models [70,71]. Furthermore, FHCL, containing these flavonoids, has demonstrated no genotoxicity in this study and has shown no acute or sub-chronic toxicity in animal studies or healthy human volunteers, thereby supporting its safety [40,72].

Environmental carcinogens typically require metabolic activation by xenobiotic-metabolizing enzymes to manifest their genotoxic effects. For example, DEN and DMH are primarily metabolized by the Phase I enzyme CYP2E1 [53], while AFB_1_ is processed by CYP1A2 and CYP3A [73]. Similarly, MeIQ is metabolized by CYP1A1 and CYP1A2 [74]. Exposure to these carcinogens has been reported to increase the expression of Phase I enzymes such as CYP2E1 and CYP1A, mediated by key regulatory proteins and receptors including pregnane X receptor (PXR), chimeric antigen receptor (CAR), aryl hydrocarbon receptor (AhR), and cytochrome P450 reductase (CPR) [53,75]. Concurrently, these carcinogens often reduce the activity of Phase II enzymes like UGT and GST, which are essential for detoxification [53,76]. In this study, FHCL was found to specifically affect indirect mutagens, which require metabolic activation by xenobiotic-metabolizing enzymes. The crude extract and DCM fraction of FHCL, known for their significant anti-genotoxic properties in in vitro models, contain key compounds such as vanillic acid, p-coumaric acid, quercetin, and quercitrin. Vanillic acid notably reduced the expression levels of Phase I enzymes, including CYP1A2 and CYP2E1, and enhanced the expression of detoxification system genes such as glutathione S-transferase alpha 5 (GSTA-5) and nuclear factor erythroid 2-related factor 2 (Nrf2) [77,78]. Quercitrin also inhibited CYP450 activities [79]. Likewise, p-coumaric acid decreased CYP1A1 enzyme activity, while quercetin modulated various Phase I and II enzymes, including CYP1A2, CYP3A4, and UGT, through mRNA expression and enzyme activities [80,81]. These findings are supported by the rat model results, which show decreased activities of Phase I enzymes CYP1A2, CYP2E1, and CYP3A2 and increased Phase II enzyme GST following a 40-day treatment with crude FHCL. Although CYP1A1 and UGT did not show significant differences, a trend toward modulation was observed. However, the potential impact of FHCL on the biotransformation system raises concerns about its interaction with other drugs metabolized by Phase I and II enzymes. Further research is required to fully understand these interactions and evaluate the broader implications of FHCL when used in conjunction with other pharmaceuticals.

In a combined-carcinogen treatment model, FHCL was found to reduce the formation of preneoplastic lesions in both the liver and colon. However, its impact on the progression of these lesions was significantly pronounced only in the liver, potentially due to the high concentration of xenobiotic-metabolizing enzymes in this organ [82]. Controlling inflammatory cells has been shown to inhibit the development of preneoplastic cancer cells [83]. FHCL and its fractions, particularly the crude and DCM fractions, demonstrated the potential to reduce NO production from macrophages, which are key inflammatory cells within tumor microenvironments [84]. Compounds like vanillic acid, p-coumaric acid, quercetin, and quercitrin, which are predominantly found in the crude and DCM fractions, have also been reported to reduce inflammation by inhibiting NO production from inflammatory cells [85,86,87,88]. This inhibition can disrupt pathways that promote cell proliferation and inhibit apoptosis within the tumor microenvironment [89]. These findings align with the observation that crude FHCL, which strongly reduced NO production, also significantly decreased cell proliferation in rat colon and liver tissues. Although this study did not find a significant effect of FHCL on cell apoptosis in preneoplastic lesions in either the liver or colon, previous studies have suggested that FHCL has the potential to induce apoptosis [37]. The lack of observable apoptotic activity in this study might be attributed to the use of a combined carcinogen treatment model, which tends to induce more severe effects compared to models involving single carcinogens [18].

Combining various compounds has been shown to enhance the activity of pharmaceuticals and mitigate the side effects of individual compounds [90]. These findings align with the observation that the crude FHCL extract demonstrates greater effectiveness in reducing inflammation and genotoxicity in vitro compared to individual fractions despite containing lower concentrations of phytochemicals. This superior performance is due to the synergistic interactions among the diverse phytochemical components in the crude extract, which supports its use in animal models. Not surprisingly, the crude extract significantly modulates xenobiotic-metabolizing enzymes and markedly reduces preneoplastic lesions and cell proliferation in the liver and colon within a robust animal carcinogenesis model. Furthermore, the crude extract neutralizes the potential toxicity associated with these compounds, as evidenced by the absence of genotoxicity or acute toxicity in animal studies. These findings further reinforce the safety and efficacy of the crude extract as a cancer-preventive agent.

## 5. Conclusions

This study highlights the significant chemopreventive potential of fermented *Houttuynia cordata* leaf (FHCL) against primary cancers of the liver and colon induced by combined carcinogens. The findings suggest that FHCL exerts its effects through multiple mechanisms, including controlling cell proliferation, inhibiting inflammation, preventing genotoxicity, and modulating xenobiotic-metabolizing enzymes. Importantly, FHCL has been shown to be safe in animal studies. These results emphasize the potential for FHCL to be developed as a preventive agent in cancer management. Further research is necessary to investigate the pharmacological interactions of FHCL with other compounds, especially its effects on xenobiotic-metabolizing enzymes, in order to fully understand its safety and efficacy in combination therapies.

## Figures and Tables

**Table 1:** Percentage yield and analysis of phytochemical constituents of crude ethanolic extract of FHCL and its fractions determined by spectrophotometry and HPLC.

	Crude	HEX	DCM	ETAC	nBA	Residue
**% yield**						
% yield/dried powder	17.94	2.29	0.67	1.58	2.82	8.65
% yield/crude	−	12.75	3.75	8.82	15.72	48.20
**Spectrophotometric analysis**						
**Total phenolic**						
mg GAE/g of extract	117.54 ± 2.32	82.15 ± 2.73	158.24 ± 3.90	287.21 ± 7.27	160.68 ± 0.36	37.83 ± 2.19
mg/g of dried powder	21.09	1.88	1.06	4.54	4.53	3.27
mg/g of crude	−	10.47	5.93	25.33	25.26	18.23
**Total flavonoid**						
mg CE/g	92.41 ± 4.07	81.41 ± 2.55	121.43 ± 5.53	263.60 ± 8.22	126.91 ± 4.62	26.09 ± 2.81
mg/g of dried powder	16.58	1.86	0.81	4.16	3.58	2.26
mg/g of crude	−	10.38	4.55	23.25	19.95	12.58
**Total hydrolysable tannin**						
mg MGE/g	22.59 ± 4.22	25.45 ± 3.47	30.27 ± 4.25	128.44 ± 18.40	72.68 ± 10.72	14.50 ± 3.00
mg/g of dried powder	4.05	0.58	0.20	2.03	2.05	1.25
mg/g of crude	−	3.24	1.14	11.33	11.43	6.99
**HPLC analysis**						
**Protocatechuic acid**						
mg/g of extract	ND	ND	7.96 ± 0.01	19.74 ± 0.02	ND	ND
mg/g of dried powder	−	−	0.053	0.312	−	−
mg/g of crude	−	−	0.299	1.741	−	−
**Chlorogenic acid**						
mg/g of extract	ND	ND	ND	32.85 ± 0.13	ND	ND
mg/g of dried powder	−	−	−	0.519	−	−
mg/g of crude	−	−	−	2.897	−	−
**Vanillic acid**						
mg/g of extract	ND	ND	12.70 ± 0.02	ND	ND	ND
mg/g of dried powder	−	−	0.009	−	−	−
mg/g of crude	−	−	0.048	−	−	−
***p*** **-Coumaric acid**						
mg/g of extract	ND	ND	13.54 ± 0.04	ND	ND	ND
mg/g of dried powder	−	−	0.091	−	−	−
mg/g of crude	−	−	0.508	−	−	−
**Rutin**						
mg/g of extract	3.59 ± 0.01	ND	ND	55.94 ± 0.01	11.72 ± 0.01	ND
mg/g of dried powder	0.644	−	−	0.884	0.331	−
mg/g of crude	−	−	−	4.934	1.842	−
**Quercetin**						
mg/g of extract	0.49 ± 0.01	0.31 ± 0.00	57.41 ± 0.00	16.36 ± 0.00	0.34 ± 0.00	ND
mg/g of dried powder	0.088	0.007	0.385	0.258	0.010	−
mg/g of crude	−	0.040	2.153	1.443	0.053	−
**Quercitrin**						
mg/g of extract	0.28 ± 0.01	ND	0.16 ± 0.01	0.26 ± 0.01	0.24 ± 0.02	ND
mg/g of dried powder	0.050	−	0.001	0.004	0.007	−
mg/g of crude	−	−	0.006	0.023	0.038	−

The spectrophotometry and HPLC results are presented as mean ± standard deviation (SD) values (*n* = 3). The mg/g of dried powder and crude extract was also calculated for the spectrophotometry and HPLC results. ND: Not Detectable; −: Not Calculated; GAE: Gallic Acid Equivalents; CE: Catechin Equivalents; MGE: Methyl Gallate Equivalents; HEX: Hexane Fraction; DCM: Dichloromethane Fraction; ETAC: Ethyl Acetate Fraction, nBA: Butanol Fraction.

**Table 2:** Genotoxicity of FHCL crude ethanolic extract and its fractions using *Salmonella* mutation assay.

Sample	Dose (mg/Plate)	Average of His^+^ Revertant Colonies per Plate (MI)			
		TA98		TA100	
		+S9	−S9	+S9	−S9
DMSO		28.4 ± 3.6	29.1 ± 0.3	127.3 ± 22.6	122.4 ± 4.7
2-AA	0.0005	840.7 ± 70.5 (29.5)	−	1496.3 ± 17.2 (12.4)	−
AF-2	0.0001	−	337.6 ± 15.6 (11.6)	−	−
AF-2	0.00001	−	−	−	842.2 ± 25.0 (6.9)
Crude	0.2	38.1 ± 0.7 (1.3)	31.8 ± 1.9 (1.1)	127.2 ± 18.4 (1.0)	127.0 ± 3.7 (1.0)
	1	76.3 ± 3.6 (2.7)	65.8 ± 1.8 (2.3)	166.7 ± 19.9 (1.3)	135.2 ± 13.0 (1.1)
	5	186.9 ± 1.6 (6.6)	152.3 ± 2.8 (5.2)	271.2 ± 33.3 (2.2)	159.4 ± 7.4 (1.3)
HEX	0.2	33.3 ± 1.2 (1.1)	36.2 ± 3.6 (1.2)	133.0 ± 13.1 (1.1)	125.0 ± 8.7 (1.0)
	1	49.7 ± 1.7 (1.8)	46.7 ± 1.9 (1.6)	166.2 ± 19.7 (1.3)	115.8 ± 2.5 (1.0)
	5	139.6 ± 16.5 (4.9)	84.3 ± 15.1 (2.9)	211.2 ± 19.7 (1.7)	131.7 ± 8.2 (1.1)
DCM	0.2	170.3 ± 7.5 (6.0)	73.3 ± 2.6 (2.5)	243.5 ± 53.8 (1.9)	163.2 ± 4.5 (1.3)
	1	382.1 ± 15.7 (13.5)	180.0 ± 13.1 (6.2)	340.3 ± 21.0 (2.8)	197.6 ± 7.5 (1.6)
	5	715.6 ± 11.2 (25.2)	311.3 ± 24.5 (10.7)	420.3 ± 0.8 (3.5)	250.4 ± 15.7 (2.0)
ETAC	0.2	141.3 ± 9.8 (5.0)	79.8 ± 5.7 (2.7)	240.3 ± 12.3 (2.0)	198.8 ± 2.2 (1.6)
	1	456.0 ± 8.7 (16.1)	238.1 ± 12.5 (8.2)	380.3 ± 34.0 (3.1)	241.3 ± 19.6 (2.0)
	5	827.1 ± 14.3 (29.1)	390.8 ± 32.3 (13.4)	459.3 ± 11.4 (3.8)	254.6 ± 7.7 (2.1)
nBA	0.2	30.7 ± 1.1 (1.1)	28.1 ± 1.1 (1.0)	112.5 ± 17.6 (0.9)	123.7 ± 5.3 (1.0)
	1	34.6 ± 1.1 (1.2)	37.2 ± 4.2 (1.3)	116.7 ± 5.7 (1.0)	128.8 ± 12.2 (1.0)
	5	50.7 ± 3.2 (1.8)	78.6 ± 2.2 (2.7)	146.8 ± 17.8 (1.2)	145.7 ± 2.4 (1.2)
Residue	0.2	26.4 ± 2.1 (0.9)	29.7 ± 1.5 (1.1)	97.7 ± 10.1 (0.8)	111.0 ± 3.7 (1.0)
	1	29.6 ± 0.9 (1.0)	31.2 ± 2.4 (1.1)	108.2 ± 13.2 (0.9)	120.0 ± 8.2 (1.0)
	5	31.0 ± 1.2 (1.1)	45.6 ± 2.5 (1.6)	126.5 ± 21.9 (1.0)	125.9 ± 5.1 (1.0)

Values are expressed as mean ± SD. MI: mutagenic index; 2-AA: 2-aminoanthracene; AF-2: 2-(2-furyl)-3-(5-nitro-2-furyl)acrylamide; HEX: hexane fraction; DCM: dichloromethane fraction; ETAC: ethyl acetate fraction; nBA: butanol fraction.

**Table 3:** Antimutagenicity against direct- and indirect-acting mutagens of crude ethanolic extract of FHCL and its fractions using salmonella mutation assay.

Sample	Dose (mg/Plate)	Average of His^+^ Revertant Colonies per Plate (%Inhibition)			
		TA98		TA100	
		+S9	−S9	+S9	−S9
DMSO		29.2 ± 8.2 ^a^	13.2 ± 3.2 ^a^	125.1 ± 16.7 ^a^	116.4 ± 7.2 ^a^
AFB_1_	0.00005	1034.9 ± 101.67	−	−	−
AF-2	0.0001	−	385.0 ± 25.5	−	−
MeIQ	0.000025	−	−	1185.8 ± 94.7	−
NaN_3_	0.001	−	−	−	526.4 ± 57.5
Crude	0.04	893.1 ± 100.9 (13.7) ^b^	325.8 ± 24.5 (16.4) ^b^	954.4 ± 6.69 (20.7) ^b^	498.1 ± 45.1 (6.2) ^b^
	0.2	419.0 ± 107.4 (57.3) ^a,b^	329.8 ± 20.6 (15.1) ^b^	477.8 ± 29.6 (66.4) ^a,b^	515.3 ± 65.2 (3.6) ^b^
	1	128.9 ± 31.8 (88.1) ^a,b^	340.7 ± 23.0 (12.3) ^b^	242.0 ± 32.1 (89.3) ^a,b^	506.7 ± 64.0 (5.6) ^b^
	5	178.7 ± 53.6 (87.1) ^a,b^	324.1 ± 12.3 (15.3) ^b^	246.0 ± 57.8 (88.8) ^a,b^	420.0 ± 34.5 (23.8) ^b^
HEX	0.04	737.8 ± 104.9 (28.7) ^b^	346.5 ± 12.7 (10.0) ^b^	779.1 ± 33.4 (34.3) ^a,c^	473.8 ± 29.3 (13.0) ^b^
	0.2	64.4 ± 4.8 (96.4) ^a,c^	336.9 ± 24.4 (13.4) ^b^	250.9 ± 16.9 (88.1) ^a,c^	473.6 ± 55.5 (13.4) ^b^
	1	48.6 ± 8.9 (97.9) ^a,b^	324.9 ± 32.8 (15.6) ^b^	196.8 ± 57.5 (93.8) ^a,b^	407.1 ± 49.5 (29.4) ^b^
	5	96.4 ± 27.3 (92.7) ^a,b^	321.9 ± 48.0 (16.4) ^b^	161.7 ± 56.9 (96.0) ^a,b^	429.8 ± 31.1 (22.7) ^b^
DCM	0.04	343.6 ± 3.3 (66.8) ^a,c^	273.4 ± 23.3 (29.0) ^b^	579.9 ± 62.5 (51.1) ^a,d^	500.1 ± 44.7 (5.0) ^b^
	0.2	135.4 ± 14.6 (88.9) ^a,c^	301.6 ± 34.6 (23.7) ^b^	219.6 ± 25.8 (90.5) ^a,c^	492.7 ± 56.5 (8.6) ^b^
	1	274.7 ± 48.9 (74.8) ^a,c^	322.3 ± 20.5 (17.3) ^b^	274.9 ± 62.6 (78.8) ^a,c^	448.7 ± 31.8 (17.7) ^b^
	5	572.0 ± 52.1 (42.1) ^a,c^	336.0 ± 32.7 (13.4) ^b^	348.6 ± 70.7 (73.1) ^a,c^	430.1 ± 31.1 (21.6) ^b^
ETAC	0.04	924.2 ± 45.7 (10.7) ^b^	350.4 ± 12.7 (9.0) ^b^	750.4 ± 35.5 (36.7) ^a,c^	452.2 ± 62.2 (14.1) ^b^
	0.2	488.7 ± 30.1 (51.6) ^a,b^	353.3 ± 19.8 (8.6) ^b^	445.0 ± 86.5 (64.7) ^a,b^	428.4 ± 33.3 (22.8) ^b^
	1	397.1 ± 6.9 (60.5) ^a,c^	374.2 ± 26.6 (3.0) ^b^	328.9 ± 35.3 (76.5) ^a,c^	419.6 ± 36.9 (25.4) ^b^
	5	532.8 ± 14.0 (46.0) ^a,c^	378.5 ± 38.6 (1.7) ^b^	370.2 ± 90.1 (67.1) ^a,c^	429.8 ± 64.1 (22.7) ^b^
nBA	0.04	877.7 ± 36.7 (15.1) ^b^	377.3 ± 30.0 (2.0) ^b^	957.9 ± 62.2 (19.2) ^b^	495.9 ± 29.3 (6.8) ^b^
	0.2	875.8 ± 110.2 (13.4) ^d^	375.1 ± 34.0 (2.7) ^b^	1124.4 ± 87.5 (4.9) ^d^	490.0 ± 43.8 (8.0) ^b^
	1	671.8 ± 28.7 (30.0) ^a,d^	338.4 ± 38.7 (12.7) ^b^	775.8 ± 53.1 (37.8) ^a,d^	450.2 ± 38.2 (16.8) ^b^
	5	195.3 ± 39.46 (86.5) ^a,b^	304.3 ± 55.2 (22.5) ^b^	319.7 ± 62.8 (82.3) ^a,b^	454.4 ± 55.8 (16.2) ^b^
Residue	0.04	1004.5 ± 103.1 (2.9) ^b^	374.2 ± 31.8 (3.2) ^b^	1148.7 ± 75.9 (3.1) ^b^	483.8 ± 55.6 (11.1) ^b^
	0.2	1022.2 ± 190.2 (1.0) ^d^	370.8 ± 26.6 (3.9) ^b^	956.0 ± 75.9 (19.3) ^d^	486.7 ± 69.5 (10.0) ^b^
	1	886.2 ± 106.7 (14.4) ^d^	370.0 ± 22.1 (4.0) ^b^	959.8 ± 53.8 (19.7) ^e^	507.8 ± 68.4 (5.6) ^b^
	5	914.7 ± 160.0 (11.3) ^d^	370.0 ± 38.3 (4.0) ^b^	1026.2 ± 91.7 (13.5) ^d^	436.7 ± 55.1 (20.8) ^b^

The values are expressed as the mean ± standard deviation. “^a^” indicates a significant difference from the mutagen control (*p* < 0.05). “^b–e^” indicate a significant difference (*p* < 0.05) between the fraction within the same dose and conditions. AFB_1_: aflatoxins B_1_. AF-2: 2-(2-furyl)-3-(5-nitro-2-furyl)acrylamide. MelQ: 2-amino-3,4-dimethylimidazo[4,5-f]quinoline. NaN_3_: sodium azide. HEX: hexane fraction. DCM: dichloromethane fraction. ETAC: ethyl acetate fraction. nBA: butanol fraction.

## Data Availability

The original contributions presented in the study are included in the article/Supplementary Material, further inquiries can be directed to the corresponding author.

## Supplementary Materials

The following supporting information can be downloaded at https://www.mdpi.com/article/10.3390/foods13223645/s1, Table S1: IC_20_ of crude ethanolic extract of FHCL and its fractions in Raw 264.7 cell lines.
