# Supplementary material for: Genotoxic and Anti-Genotoxic Assessments of Fermented Houttuynia cordata Thunb. Leaf Ethanolic Extract and Its Anti-Cancer Effect in a Dual-Organ Carcinogenesis Model of Colon and Liver in Rats
Source: Foods. 2024 Nov 15;13(22):3645. doi: 10.3390/foods13223645 (PMC11594090; doi:10.3390/foods13223645)
Supplement: Supplementary file 1 [file foods-13-03645-s001.zip › foods-3271835-supplementary.pdf]

Genotoxic and Anti-Genotoxic Assessments of Fermented *Houttuynia cordata* Thunb. Leaf Ethanolic Extract and Its Anti-Cancer Effect in a Dual-Organ Carcinogenesis Model of Colon and Liver in Rats

Chonikarn Singai [1], Pornsiri Pitchakarn [1], Sirinya Taya [2], Rawiwan Wongpoomchai [1] and Ariyaphong Wongnoppavich [1,*]

[1] Department of Biochemistry, Faculty of Medicine, Chiang Mai University, Chiang Mai 50200, Thailand
[2] Functional Food Research Unit, Multidisciplinary Research Institute, Chiang Mai University, Chiang Mai 50200, Thailand
* Correspondence: ariyaphong.w@cmu.ac.th

Supplementary Table S1. IC_{20} of crude ethanolic extract of FHCL and its fractions in Raw 264.7 cell lines.

**Sample**	**IC_{20} (μg/ml)**
Crude	273.12 ± 21.43
HEX	127.09 ± 19.99
DCM	144 ± 25.56
EAC	≥500
BA	≥500
Residue	≥500
